# Effects of perioperative goal-directed fluid therapy combined with the application of alpha-1 adrenergic agonists on postoperative outcomes: a systematic review and meta-analysis

**DOI:** 10.1186/s12871-018-0564-y

**Published:** 2018-08-17

**Authors:** Shuai Feng, Shuyi Yang, Wei Xiao, Xue Wang, Kun Yang, Tianlong Wang

**Affiliations:** 1 Department of Anesthesiology, Xuanwu Hospital, Capital Medical University, Beijing, China; 2 Department of Library, Xuanwu Hospital, Capital Medical University, Beijing, China; 3 Department of Evidence-based Medicine, Xuanwu Hospital, Capital Medical University, Beijing, China

**Keywords:** Alpha-1 adrenergic agonists, Anesthesia management, Goal-directed fluid therapy, Length of hospital stay, Noncardiac surgery, Morbidity, Mortality

## Abstract

**Background:**

Past studies have demonstrated that goal-directed fluid therapy (GDFT) may be more marginal than previously believed. However, beneficial effects of alpha-1 adrenergic agonists combined with appropriate fluid administration is getting more and more attention. This study aimed to systematically review the effects of goal-directed fluid therapy (GDFT) combined with the application of alpha-1 adrenergic agonists on postoperative outcomes following noncardiac surgery.

**Methods:**

This meta-analysis included randomized controlled trials (RCTs) on GDFT combined with the application of alpha-1 adrenergic agonists in patients undergoing noncardiac surgery. The primary outcomes included the postoperative mortality rate and length of hospital stay (LOS). The secondary outcome indexes were the incidence of postoperative complications and recovery of postoperative gastrointestinal (GI) function. The traditional pairwise meta-analysis was conducted to compare the effect of fluid therapy. The quality of included RCTs was evaluated according to the Cochrane Collaboration’s risk-of-bias tool. Also, the publication bias was detected using funnel plots, Egger’s regression test, and Begg’s adjusted rank correlation test. The meta-analysis was conducted using the RevMan 5.3 and Stata 14.0 software.

**Results:**

Thirty-two eligible RCTs were included in this meta-analysis. Perioperative GDFT combined with the application of alpha-1 adrenergic agonists was associated with a significant reduction in LOS (*P* = 0.002; *I*^2^ = 69%), and overall complication rates (*P* = 0.04; *I*^2^ = 41%). It facilitated gastrointestinal function recovery, as demonstrated by shortening the time to first flatus by 6.30 h (*P* < 0.00001; *I*^2^ = 91%) and the time to toleration of solid food by 1.69 days (*P* < 0.00001; *I*^2^ = 0%). Additionally, there was no significant reduction in short-term mortality in the GDFT combined with alpha-1 adrenergic agonists group (*P* = 0.05; *I*^2^ = 0%).

**Conclusion:**

This systematic review of available evidence suggested that the use of perioperative GDFT combined with alpha-1 adrenergic agonists might facilitate recovery in patients undergoing noncardiac surgery.

**Electronic supplementary material:**

The online version of this article (10.1186/s12871-018-0564-y) contains supplementary material, which is available to authorized users.

## Background

Perioperative fluid management has been regarded as a significant part of enhanced recovery after surgery (ERAS) pathway. It has been shown to improve outcomes following major surgery in high-risk patients [1–3]. Increasing evidence has suggested to change liberal or restrictive hydration strategy to goal-directed fluid therapy (GDFT). GDFT can be aimed at single or multiple goals, such as functional hemodynamic parameters, indexes of oxygen delivery or consumption. Perioperative GDFT which is an individualized fluid administration strategy based on different techniques, such as pulse contour analysis technique, thermodilution technique and esophageal Doppler, is related to accelerated recovery of gastrointestinal (GI) function, reduced length of hospital stay (LOS), and reduced postoperative complication rates following surgery [4]. Several systematic reviews and meta-analyses investigated that GDFT might decrease postoperative mortality and morbidity in surgical patients [5–7], but others suggested that the advantage might be more marginal than previously believed [8–10].

Many recent studies have demonstrated the beneficial effects of the infusion or injection of alpha-1 adrenergic agonists combined with appropriate fluid administration [11–13]. GDFT combined with alpha-1 adrenergic agonists, such as norepinephrine and phenylephrine, may improve postoperative outcomes following major surgery because it maintains appropriate vascular tension, blood pressure, and organ perfusion. At present, no systematic review on this aspect has been reported. Therefore, this was the first systematic review and meta-analysis to evaluate all available evidence regarding the effect of GDFT with the application of alpha-1 adrenergic agonists compared with the conventional fluid therapy on postoperative outcomes following noncardiac surgery.

## Methods

Preferred Reporting Items for Systematic Reviews and Meta-Analyses (PRISMA) guidelines were followed in reporting this systematic review and meta-analysis [14]. A review protocol was developed prior to conducting the study.

### Inclusion and exclusion criteria

The eligible studies if this systematic review and meta-analysis were identified following the patient, intervention, comparison, outcomes, and study design strategy [15].

#### Types of studies

##### Inclusion

Only randomized controlled trials (RCTs) were included.

##### Exclusion

Observational cohort and case–control studies, case reports, experimental studies, and reviews were excluded.

#### Types of participants

Adult patients (aged ≥18 years) undergoing noncardiac surgery were evaluated. Studies involving pediatric patients, patients undergoing cardiac surgery, and nonsurgical patients were excluded.

#### Types of interventions

Perioperative GDFT was used as intervention treatment, which was defined as perioperative administration (initiated before surgery or maintained during the intraoperative period, or performed in the postoperative period until 8 h) of fluids combined with the application of alpha-1 adrenergic agonists.

#### Types of outcome measurement

Primary outcome measures were mortality and LOS. Secondary outcome measures were gastrointestinal (GI) function recovery (i.e., time to tolerate oral diet, time to first flatus) and morbidity, evaluated as the number of patients with the number of postoperative complications in non-cardiac surgery. Postoperative complications included acute myocardial ischemia, severe arrhythmias, acute heart failure, postoperative hypotension, respiratory infections, respiratory support, acute kidney injury (AKI), urinary infections, ileus, postoperative nausea and vomiting (PONV), coagulation abnormalities, wound infections, and surgical complications.

### Search strategy and study selection

Published and unpublished RCTs in English language were identified from electronic databases of MEDLINE, Embase, and the Cochrane Central Register of Controlled Trials. The search strategy was drafted by an experienced librarian, and the appropriate retrieval was shown through the combination of medical subject headings and free text words. The definite search strategy used in Medical Literature Analysis and Retrieval System Online (MEDLINE) is presented in Additional file 1. Furthermore, registered trials and unpublished data were also identified by searching: (1) international trials registries, mainly including ClinicalTrials. gov and World Health Organization International Clinical Trials Registry Platform and (2) the International Prospective Register of Systematic Reviews. Additionally, relevant RCTs were obtained by hand-searching reference lists of included studies and relevant reviews. Also, corresponding authors of included RCTs and other possible institutions were contacted for unpublished trials when necessary.

**Fig. 1:**
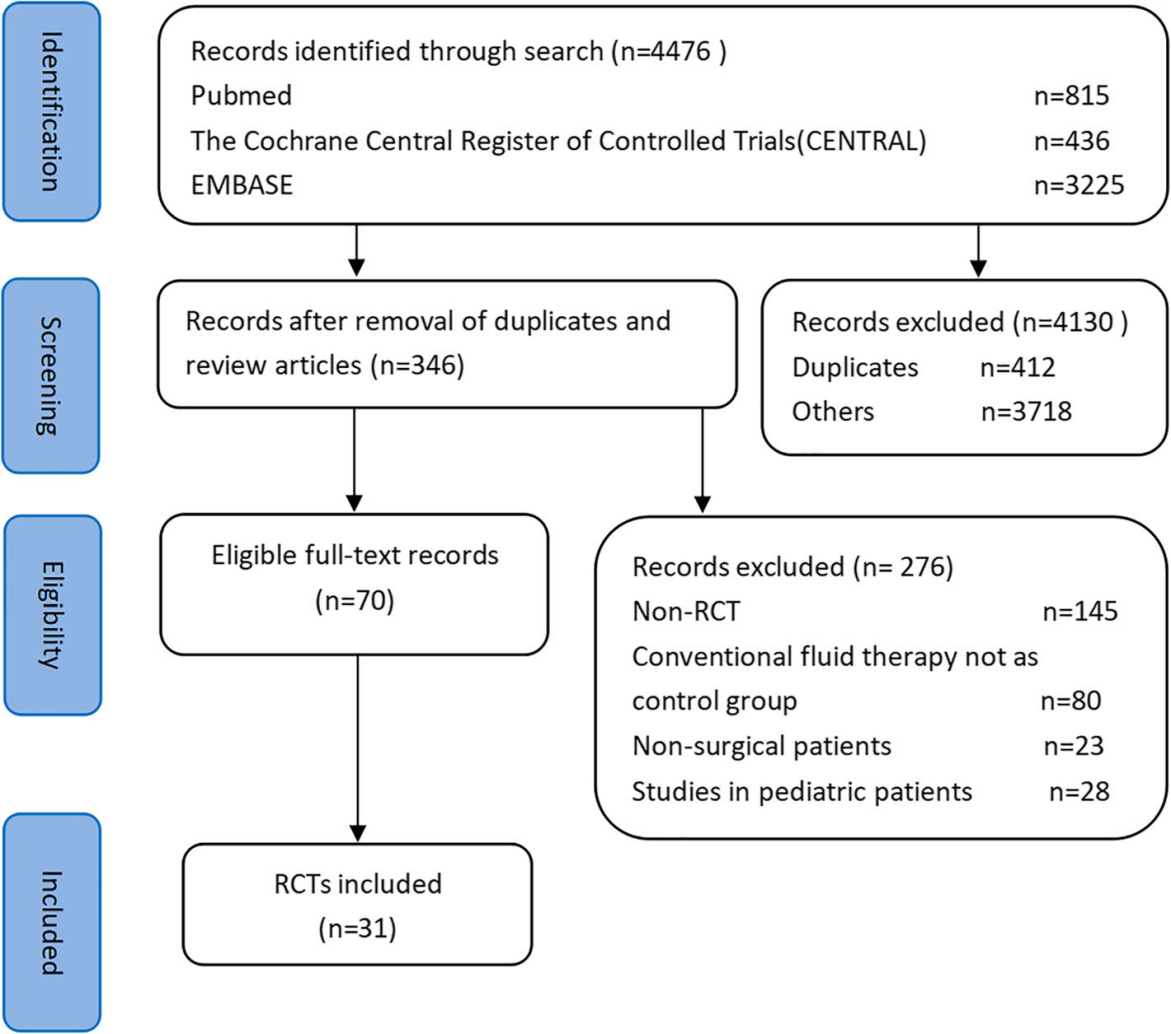
Preferred Reporting Items for Systematic Reviews and Meta-Analyses (PRISMA) diagram of study selection. RCTs, Randomized controlled trials

According to the eligibility criteria, two reviewers independently completed the two levels of study screening and selection. Disagreements between reviewers were resolved by consensus. The selection process for relevant studies retrieved from databases is shown in a PRISMA-compliant [14] flow chart (Fig. 1).

### Data extraction

The following data was extracted from every study: first author name, year of publication, sample size, type of surgery, the GDFT strategy (goals, monitoring methods, and interventions) and the type of alpha-1 adrenergic agonists. The primary endpoints of this study included mortality and LOS. The secondary outcomes were postoperative complication rates (i.e., number of patients with complications following noncardiac surgery) and recovery of GI function, including time to first flatus and time to toleration of solid food. Two reviewers (SF and SY) extracted the aforementioned data independently. Disagreements were resolved by consensus. The authors were contacted and requested for original data by email when necessary to obtain complete data and optimize further details from the studies.

### Risk-of-bias assessment

The Cochrane Collaboration’s tool [16] for assessing the risk of bias was applied independently by two reviewers. The risk of bias was evaluated as high, low, or unclear for each of selection bias, performance bias, detection bias, attrition bias, and reporting bias. Information for judging the risk of bias was collected from all reports originating from each study as well as from the protocol published in the registry. Disagreements were resolved through discussion.

Grading of Recommendations, Assessment, Development, and Evaluations (GRADE) methods were used to evaluate the quality of evidence for each outcome, classified as very low, low, moderate, or high [17, 18]. It was evaluated using GRADEPro software 3.6 (GRADE Working Group).

### Statistical analysis

Traditional pairwise meta-analysis of the included RCTs with the random-effects model [19, 20] due to the expected heterogeneity was conducted. The pooled risk ratios (RR) and 95% CI were calculated to measure the strength of the association between GDFT combined with the application of alpha-1 adrenergic agonists and conventional fluid treatment. The *Z* test was used to determine the significance of the pooled effect size, and a *P* value < 0.05 was considered statistically significant. The heterogeneity was examined using the Q test and *I*^2^ statistic. A *Q*-test *P* value of more than 0.10 showed that no heterogeneity existed among the included studies. The Mantel–Haenszel fixed-effects model was used for pooling; otherwise, the random-effects model was used. *I*^2^ statistic (between 0 and 100%), which was defined as the proportion of the observed study variability owing to heterogeneity instead of chance, was also used to evaluate the heterogeneity [21]. The I^2^value more than 40% heterogeneity was considered significant. All aforementioned statistical analyses were accomplished using Review Manager (The Cochrane Collaboration, Oxford, UK; version 5.3.5).

Publication bias was assessed using the funnel plots, Egger’s regression test [22], and Begg’s adjusted rank correlation [23], which was conducted with the Stata software (Stata Corp., TX, USA; version 14.0).

Subgroup analysis based on age of patients, types of surgeries and alpha-1 adrenergic agonists of the RCTs which were the potential sources of heterogeneity were conducted. Otherwise, we planned a sensitivity analysis dividing studies in low/unclear/high risk of bias to investigate the robustness of results.

## Results

We initially identified 4476 studies by searching the MEDLINE, Embase and the Cochrane Central Register of Controlled Trials. After title and abstract screening, 4130 citations were excluded because of duplication of published data, reviews and not reporting original research. A total of 346 RCTs were gathered for further review. Of this group, 276 studies were excluded because they were not RCTs, involved nonsurgical patients or pediatric patients, or did not use conventional fluid therapy as a control. Finally, 31 studies were considered for the present systematic review (Fig. 1).

### Study characteristics

The 31 RCTs [3, 9, 24–52] yielded 3176 patients (Table 1). Sample sizes ranged from 40 to 199. All studies were reported between 2002 and 2017.

**Table 1:** Study characteristics and overall risk-of-bias assessment for each study

Trial/author, year [reference]	Number of patients	Nature of surgery	Goal-directed hemodynamic therapy			Overall risk of bias
			Goal	Monitoring method	Type of α1 adrenergic agonists application	
Bartha et al., 2013 [24]	149	Proximal femoral fracture	DO^2^I > 600 mL/(min · m^2^), ΔSV < 10%	Pulse contour analysis monitor	Phenylephrine	High
Benes et al., 2015 [25]	80	Total knee and hip replacement	PPV < 13%	Pulse contour analysis monitor	Norepinephrine	Low
Bisgaard, et al., 2013 [26]	64	Abdominal aortic surgery	SVI < 10%, DO^2^I ≥ 600 mL/(min · m^2^)	Pulse contour analysis monitor	Phenylephrine	Low
Broch et al., 2016 [27]	79	Major abdominal surgery	PPV ≤10%; CI ≥2.5 L/(min · m^2^) MAP ≥65 mmHg	Noninvasive hemodynamic optimization	Norepinephrine	Unclear
Elgendy et al., 2017 [28]	86	Major abdominal surgery	SVV ≤12%; CI ≥2.5 L/(min · m^2^) MAP ≥65 mmHg	Pulse contour analysis monitor	Norepinephrine	Low
Forget et al., 2010 [29]	82	Major abdominal surgery	PVI < 13%	Pulse oximeter	Norepinephrine	Low
Funk et al., 2015 [30]	40	Open abdominal aortic aneurysm repair	SVV ≤13%, CI ≥2.2 L/(min · m^2^)	Pulse contour analysis monitor	Norepinephrine	Low
Gan et al., 2002 [31]	100	Major abdominal surgery	FTc > 0.40 s, ΔSV < 10%	Esophageal Doppler	Phenylephrine	High
Gómez-Izquierdo et al., 2017 [32]	128	Elective laparoscopic colorectal surgery	ΔSV < 11%	Esophageal Doppler	Phenylephrine	Low
Hand et al., 2016 [33]	94	Free tissue transfer reconstruction	SVV ≤12%; CI ≥3.0 L/(min · m^2^), MAP > 75 mmHg	Pulse contour analysis monitor	Phenylephrine	High
Kaufmann et al., 2017 [34]	96	Thoracic surgery	ΔSV < 10%; CI ≥2.5 L/(min · m^2^), MAP ≥70 mmHg	Esophageal Doppler	Norepinephrine	Low
Kumar et al., 2015 [35]	40	Major abdominal surgery	SVV ≤10%; O_2_ER < 27%, MAP > 65 mmHg	Pulse contour analysis monitor	Norepinephrine	Unclear
Luo et al., 2017 [36]	145	Brain surgery	SVV ≤15%; CI ≥2.5 L/(min · m^2^), MAP ≥65 mmHg	Pulse contour analysis monitor	Norepinephrine	Low
Malbouisson et al., 2017 [37]	168	Open major surgery	PPV ≤10%	Pulse contour analysis monitor	Norepinephrine	High
Mayer et al., 2010 [38]	60	High-risk surgical	SVV ≤12%; CI ≥2.5 L/(min · m^2^), SVI ≥35 mL/m^2^, MAP > 65 mmHg	Pulse contour analysis monitor	Norepinephrine	Low
Moppett et al., 2015 [9]	114	Hip fracture surgery	ΔSV < 10%	Pulse contour analysis monitor	Metaraminol	High
Peng et al., 2014 [39]	80	Major orthopedic surgery	SVV ≤10%	Pulse contour analysis monitor	Phenylephrine	High
Pestaña et al., 2014 [40]	142	Major abdominal surgery	CI ≥2.5 L/(min · m^2^), MAP ≥65 mmHg	Noninvasive cardiac output monitor	Norepinephrine	Low
Pösö et al., 2014 [41]	46	Laparoscopic bariatric surgery	SVV < 12%, CO, SV, and MAP > 70% baseline	Pulse contour analysis monitor	Phenylephrine	High
Reisinger et al., 2017 [42]	58	Colorectal surgery	ΔSV < 10%, MAP ≥65 mmHg	Esophageal Doppler	Phenylephrine	Low
Salzwedel et al., 2013 [43]	160	Major abdominal surgery	PPV ≤10%; CI ≥2.5 L/(min · m^2^), MAP ≥65 mmHg	Pulse contour analysis monitor	Norepinephrine, phenylephrine	High
Scheeren et al., 2013 [3]	52	High-risk surgery	SVV ≤10%, ΔSV < 10%	Pulse contour analysis monitor	Norepinephrine	High
Schmid et al., 2016 [44]	180	Major abdominal surgery	GEDI ≤800; CI ≥2.5 L/(min · m^2^), ELWI ≥10 mL/m^2^, MAP ≥70 mmHg	Transpulmonary thermodilution monitor	Norepinephrine	Low
Stens et al., 2017 [45]	175	Moderate-risk abdominal surgery	PPV < 12%, CI ≥2.5 L/(min · m^2^), MAP ≥70 mmHg	Pulse contour analysis monitor	Norepinephrine	Low
Veelo et al., 2017 [46]	199	Esophageal surgery	ΔSV < 10%, MAP ≥65 mmHg	Pulse contour analysis monitor	Norepinephrine, Phenylephrine	Unclear
Wagar et al., 2017 [47]	67	Total pancreatectomy and islet cell autotransplantation	SVV < 12%; CI ≥2.5 L/(min · m^2^); MAP > 10% baseline	Pulse contour analysis monitor	Phenylephrine	Unclear
Weinberg et al., 2017 [48]	52	Pancreaticoduodenectomy	SVV ≤20%, MAP ≥20% baseline	Pulse contour analysis monitor	Norepinephrine	Low
Wu et al., 2017 [49]	63	Supratentorial neoplasms surgery	SVV ≤12%, CI ≥2.5 L/(min · m^2^)	Pulse contour analysis monitor	Phenylephrine	Low
Xu et al., 2017 [50]	168	Elective thoracoscopic lobectomy	SVV ≤13%; CI > 2.5 L/(min · m^2^), ΔSV ≤10%, MAP > 65 mmHg	Pulse contour analysis monitor	Norepinephrine	Low
Zhang et al., 2013 [51]	60	Thoracoscopy lobectomy	SVV < 9%, CI ≥2.5 L/(min · m^2^)	Pulse contour analysis monitor	Phenylephrine	Low
Zheng et al., 2013 [52]	60	Gastrointestinal surgery	SVI ≤35 mL/m^2^; MAP > 65 mmHg; SVV < 12%; CI ≥2.5 L/(min · m^2^)	Pulse contour analysis monitor	Norepinephrine	Low

*Abbreviations*: *CI* Cardiac index, *CO* Cardiac output, *CVP* Central venous pressure, *DO*_*2*_*I* Oxygen delivery index, *ELWI* Extravascular lung water index, *FTc* Corrected flow time, *GEDI* Global end-diastolic index, *MAP* Mean arterial pressure, *O*_*2*_*ER* Oxygen extraction ratio, *PPV* Pulse pressure variation, *PVI* Pleth variability index, *SV* Stroke volume, *SVI* Stroke volume index, *SVV* Stroke volume variation

**Fig. 2:**
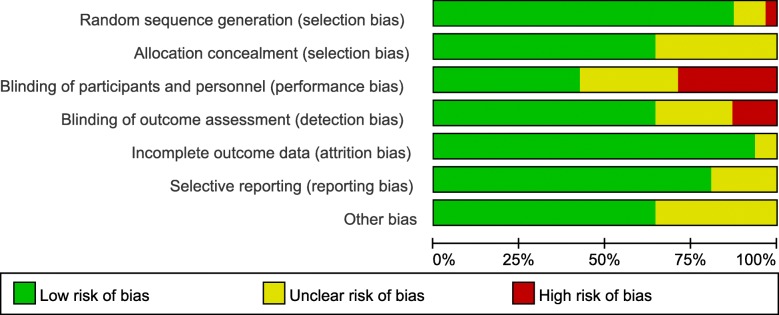
Review authors’ judgments about each risk-of-bias item presented as percentages across all included studies

The risk of bias was analyzed with the Cochrane tool. The methodological quality of included trials is presented in a summary graph (Fig. 2) and table (Additional file 2). A total of 18 studies (58%) [25, 26, 28–30, 32, 34, 36, 38, 40, 42, 44, 45, 48–52] were judged to carry a low risk of bias (Table 1).

Seventeen trials [28, 29, 31–33, 35, 36, 39, 41, 43, 45–51] included patients who were less than 65 years old, and 14 trials [3, 9, 24–27, 30, 34, 37, 38, 40, 42, 44, 52] included patients who were more than 65 years old. As for the type of alpha-1 adrenergic agonists used in studies, 17 studies [3, 25, 27–30, 34–38, 40, 44, 45, 49–52] selected norepinephrine, 11 studies [24, 26, 31–33, 39, 41, 42, 50, 52] considered phenylephrine, 2 studies [43, 46] used norepinephrine and phenylephrine, and 1 study [9] used metaraminol. Additionally, 18 trials [3, 27–29, 31, 32, 35, 37, 38, 40–45, 47, 48, 52] involved abdominal surgery. Four trials [34, 46, 50, 51] were related to thoracic surgery. Three trials [9, 24, 25] were about orthopedic surgery. Six trials [26, 30, 33, 36, 39, 49] were about other kinds of surgery.

### Meta-analyses

#### Short-term mortality

**Fig. 3:**
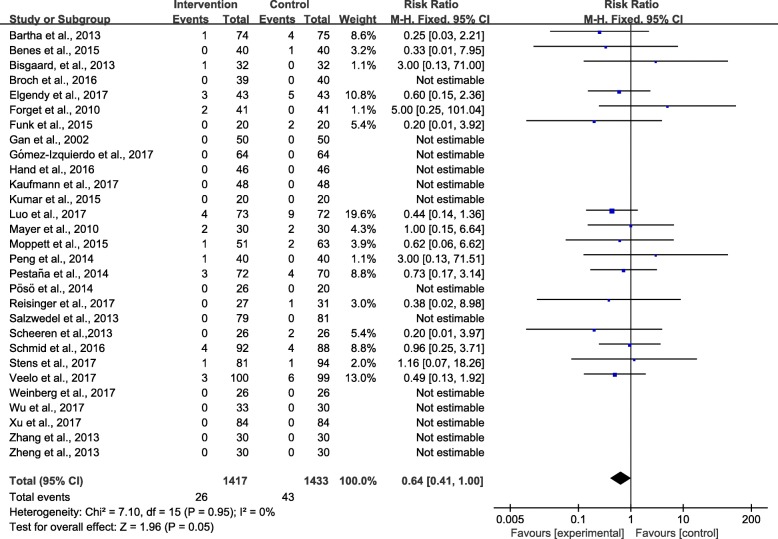
Meta-analysis and pooled risk ratio (RR) of the effect of goal-directed fluid therapy (GDFT) combined with alpha-1 adrenergic agonists on the short-term mortality after noncardiac surgery

Twenty-nine studies [3, 9, 24–36, 38–46, 48–52] provided suitable data for the meta-analysis. The pooled short-term mortality was 26 (1.8%) of 1417 in the intervention group and 43 (3%) of 1433 in the control group, and the RR was 0.64 (95% CI 0.41–1.00; *P* = 0.05; *I*^2^ = 0%), showing no significant reduction in mortality in the GDFT combined with alpha-1 adrenergic agonists group (Fig. 3). The influence analysis of individual studies on the pooled RR is presented in Additional file 3. The GRADE quality of evidence was judged to be high. A funnel plot is presented in Additional file 4. Neither Egger’s regression asymmetry test (*P* = 0.465) nor Begg’s adjusted rank correlation test (*P* = 0.471) showed any evidence of publication bias regarding short-term mortality.

#### Length of hospital stay

**Fig. 4:**
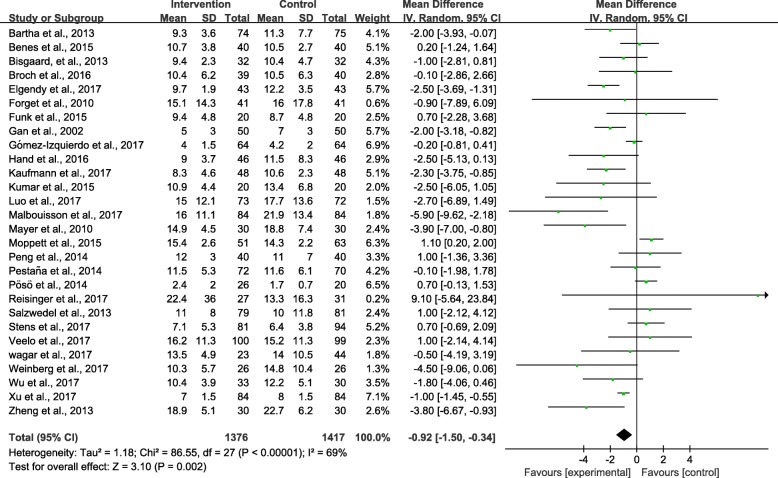
Meta-analysis and pooled risk ratio (RR) of the effect of goal-directed fluid therapy (GDFT) combined with alpha-1 adrenergic agonists on the length of hospital stay (LOS) after noncardiac surgery

Twenty-eight studies [9, 24–43, 45–50, 52] provided data for analysis. Perioperative GDFT combined with alpha-1 adrenergic agonists shortened the LOS [weighted mean difference (WMD) –0.92 days; 95% confidence interval (CI) –1.50 to − 0.34; *P* = 0.002; *I*^2^ = 69%] (Fig. 4). The influence analysis of individual studies on the pooled RR is presented in Additional file 5. The GRADE quality of evidence was judged to be moderate, downgraded for inconsistency. A funnel plot is presented in Additional file 6. Neither the Egger’s regression asymmetry test (*P* = 0.591) nor the Begg’s adjusted rank correlation test (*P* = 0.750) showed any evidence of publication bias regarding short-term mortality.

**Fig. 5:**
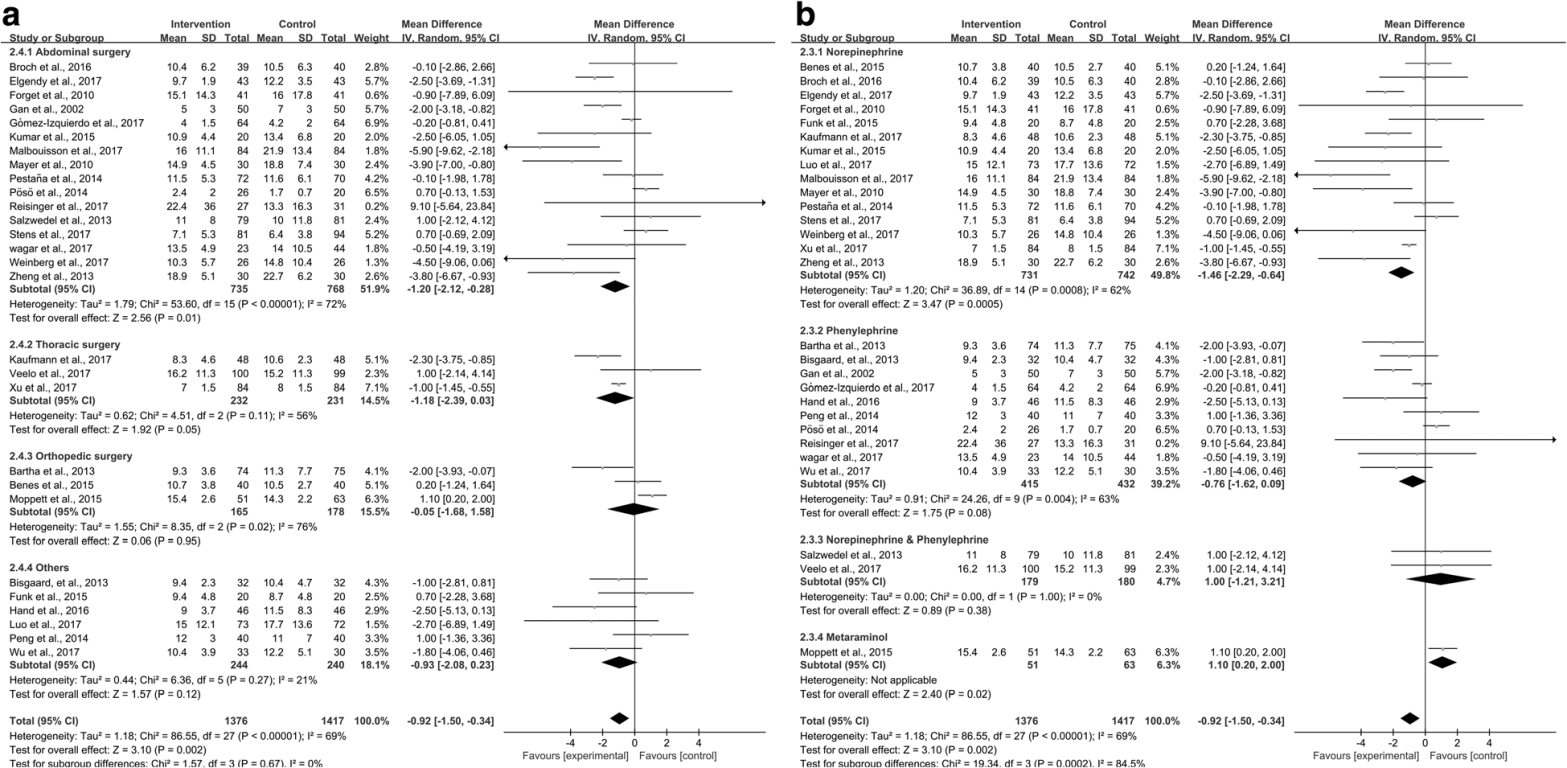
Forest plot comparing the length of hospital stay (LOS) for patients receiving GDFT combined with alpha-1 adrenergic agonists versus control divided by the (**a**) type of surgery: abdominal surgery, thoracic surgery, orthopedic surgery, or other surgery and (**b**) type of alpha-1 adrenergic agonists: norepinephrine, phenylephrine, norepinephrine combined with phenylephrine, or metaraminol

The subgroup analyses revealed that GDFT combined with alpha-1 adrenergic agonists significantly reduced LOS associated with abdominal surgery (WMD − 1.20 days; 95% CI –2.12 to − 0.28; *P* = 0.01; *I*^2^ = 71%; *n* = 16 [3, 27–29, 31, 32, 35, 38, 40–45, 48, 52]), norepinephrine as interventions (WMD − 1.46 days; 95% CI –2.29 to − 0.64; *P* = 0.0005; *I*^2^ = 62%; *n* = 16 [3, 25, 27–30, 34–36, 38, 40, 44, 45, 48, 50, 52]) (Fig. 5) and low risk of bias (WMD − 0.98 days; 95% CI –1.58 to − 0.38; *P* = 0.001; *I*^2^ = 56%; *n* = 16 [25, 26, 28–30, 32, 34, 36, 38, 40, 42, 45, 48–50, 52]) (Additional file 7).

#### GI function recovery

**Fig. 6:**
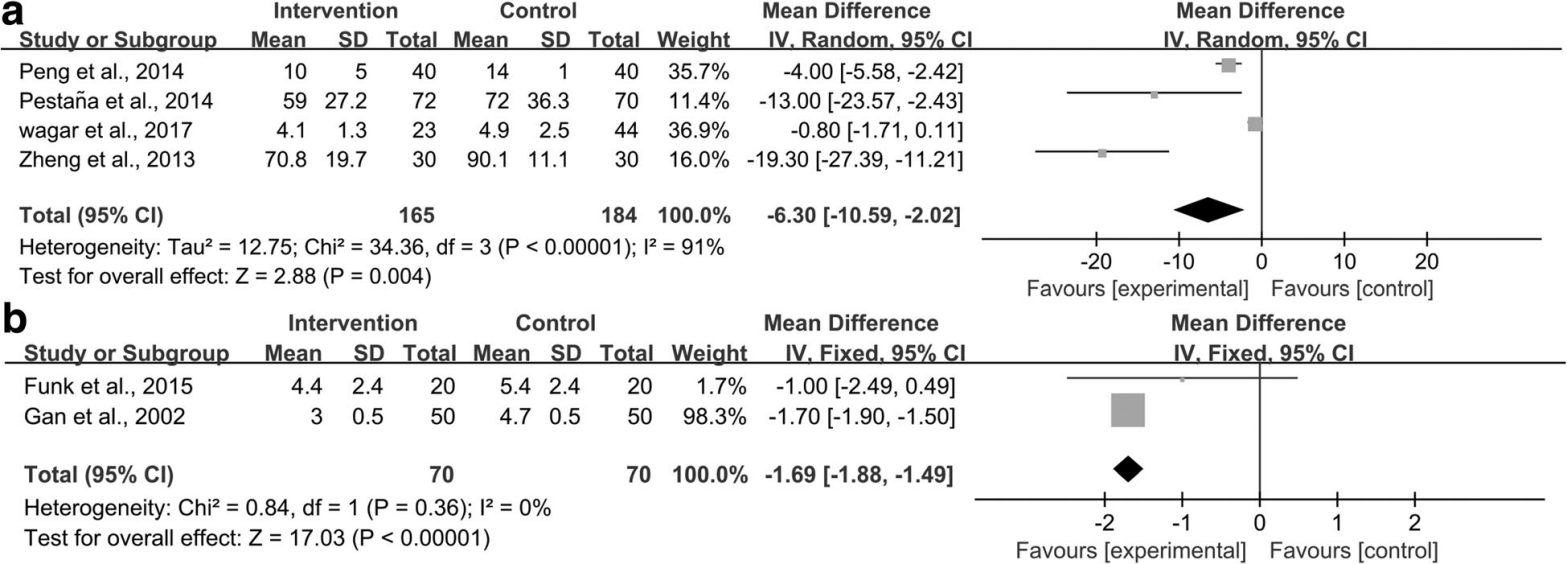
Meta-analysis and pooled risk ratio (RR) of the effect of goal-directed fluid therapy (GDFT) combined with alpha-1 adrenergic agonists on gastrointestinal (GI) function recovery after noncardiac surgery and the influence analysis of individual studies on the pooled RR. Forest plots for (**a**) time to first flatus and (**b**) time to toleration of solid food

Perioperative GDFT combined with alpha-1 adrenergic agonists shortened the time to first flatus (WMD − 6.30 h; 95% CI –10.59 to − 2.20; *P* = 0.004; *I*^2^ = 91%; *n* = 4 [39, 40, 47, 52]) and time to toleration of solid food (WMD − 1.69 days; 95% CI –1.88 to − 1.49; *P* < 0.00001; *I*^2^ = 0%; *n* = 2 [30, 31]) (Fig. 6). The GRADE quality of time to first flatus was judged to be low, downgraded by inconsistency, while the GRADE quality of time to toleration of solid food was judged to be high. The subgroup analyses based on the age of patients, type of surgery, and type of alpha-1 adrenergic agonists were not performed owing to the limited number of studies.

#### Postoperative complications

**Fig. 7:**
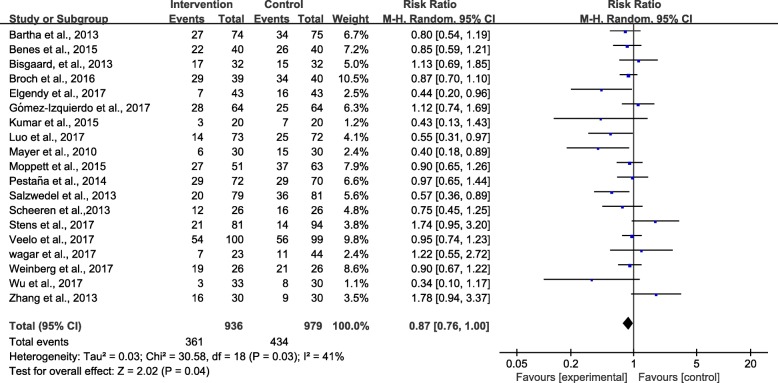
Meta-analysis and pooled risk ratio (RR) of the effect of goal-directed fluid therapy (GDFT) combined with alpha-1 adrenergic agonists on overall complication rates after noncardiac surgery and the influence analysis of individual studies on the pooled RR

Nineteen trials [3, 9, 24–28, 32, 35, 36, 38, 40, 43, 45–49, 51] reported suitable data on the numbers of patients with complications. The pooled RR of 0.87 showed reduced overall complication rates after surgery in the GDFT group compared with the control group (95% CI 0.76–1.00; *P* = 0.04; *I*^2^ = 41%) (Fig. 7). The influence analysis of individual studies on the pooled RR is presented in Additional file 8. The GRADE quality of evidence was judged to be high. A funnel plot is presented in Additional file 9 Neither the Egger’s regression asymmetry test (*P* = 0.137) nor the Begg’s adjusted rank correlation test (*P* = 0.206) showed evidence of publication bias regarding overall complication rates.

The subgroup analyses showed a significant reduction in the intervention group in studies including patients ≥65 years old (RR 0.86; 95% CI 0.75–0.98; *P* = 0.02; *I*^2^ = 0%; *n* = 9 [3, 9, 24–27, 35, 38, 40]) and using norepinephrine as an intervention (RR 0.81; 95% CI 0.66–0.99; *P* = 0.04; *I*^2^ = 45%; *n* = 10 [3, 25, 27, 28, 35, 36, 38, 40, 45, 48]) (Additional file 10).

**Fig. 8:**
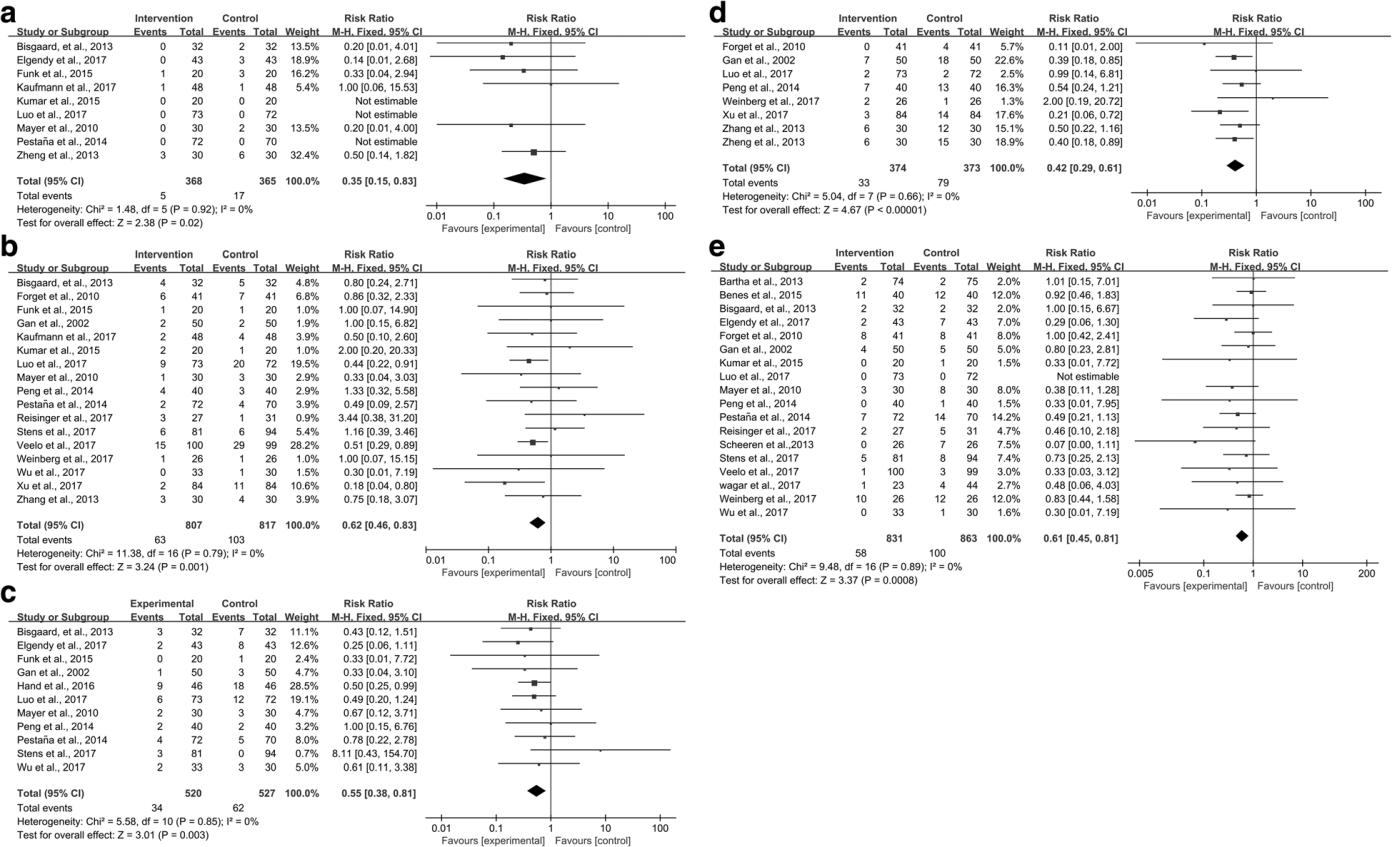
Meta-analysis and pooled risk ratio (RR) of the effect of goal-directed fluid therapy (GDFT) combined with alpha-1 adrenergic agonists on postoperative complications after noncardiac surgery and the influence analysis of individual studies on the pooled RR. Forest plots for (**a**) postoperative myocardial ischemia, (**b**) postoperative respiratory infection, (**c**) postoperative respiratory support, (**d**) postoperative nausea and vomiting (PONV), and (**e**) wound infection

As for postoperative cardiovascular complications, 9 trials [26, 28, 30, 34–36, 38, 40, 52] reported suitable data on the numbers of patients with postoperative myocardial ischemia. The pooled RR of 0.35 showed a reduction in this complication rate after surgery in the intervention group compared with the control group (95% CI 0.15–0.83; *P* = 0.02; *I*^2^ = 0%) (Fig. 8a). The GRADE quality of evidence was judged to be high. Neither the Egger’s regression asymmetry test (*P* = 0.274) nor the Begg’s adjusted rank correlation test (*P* = 0.452) showed evidence of publication bias. Postoperative heart failure [36, 40, 54], arrhythmia [26, 30, 34–36, 38, 40, 48, 49, 52], and hypotension [35, 38–40, 43, 45, 49, 50] showed no significant difference between the intervention and control groups.

As for postoperative respiratory complications, 17 trials [26, 29–31, 34–36, 38–40, 42, 45, 46, 48–51] reported suitable data on the numbers of patients with postoperative respiratory infection. The pooled RR of 0.62 showed a reduction in this complication rate after surgery in the intervention group compared with the control group (95% CI 0.46–0.83; *P* = 0.001; *I*^2^ = 0%) (Fig. 8b). Neither the Egger’s regression asymmetry test (*P* = 0.236) nor the Begg’s adjusted rank correlation test (*P* = 0.762) showed any evidence of publication bias. Eleven trials [26, 28, 30, 31, 33, 36, 38–40, 45, 49] reported suitable data on the numbers of patients with postoperative respiratory support. The pooled RR of 0.55 showed a reduction in this complication rate following surgery in the intervention group compared with the control group (95% CI 0.38–0.81; *P* = 0.003; *I*^2^ = 0%) (Fig. 8c). The GRADE quality of both postoperative respiratory infection and respiratory support was judged to be high. Neither the Egger’s regression asymmetry test (*P* = 0.329) nor the Begg’s adjusted rank correlation test (*P* = 0.533) showed evidence of publication bias.

As for postoperative gastrointestinal complications, eight trials [29, 31, 36, 39, 48, 50–52] reported suitable data on the numbers of patients with PONV. The pooled RR of 0.42 showed a reduction in this complication rate after surgery in the intervention group compared with the control group (95% CI 0.29–0.61; *P* = 0.00001; *I*^2^ = 0%) (Fig. 8d). The GRADE quality of evidence was judged to be high. Neither the Egger’s regression asymmetry test (*P* = 0.792) nor the Begg’s adjusted rank correlation test (*P* = 0.536) showed any evidence of publication bias. Postoperative ileus [30, 35, 38, 40, 42, 48, 52] showed no significant difference between the intervention and control groups.

As for other complications, 18 trials [3, 24–26, 28, 29, 31, 35, 36, 38–40, 42, 45–49] reported data on the numbers of patients with postoperative wound infection. The pooled RR of 0.61 showed a reduction in this complication rate following surgery in the intervention group compared with the control group (95% CI 0.45–0.81; *P* = 0.0008; *I*^2^ = 0%) (Fig. 8e). The Begg’s adjusted rank correlation test (*P* = 0.130) showed no evidence of publication bias, whereas the Egger’s regression asymmetry test (*P* = 0.006) showed a different result. The GRADE quality of evidence was judged to be high. Other postoperative complications, including AKI [9, 24, 26, 30, 31, 34–36, 38–40, 44, 48, 50], urinary infection [24, 26, 35, 42, 45], neurologic complications [9, 24–26, 30, 34, 36, 38–40, 44–46, 48, 49], coagulation complications [25, 29, 31], and surgical complications [26, 28–30, 32, 33, 35, 38–40, 42, 44–48, 52], showed no significant difference between the intervention and control groups.

## Discussion

This systematic review and meta-analysis found that GDFT combined with alpha-1 adrenergic receptor agonists reduced LOS and overall complication rates. It also facilitated GI functional recovery, as demonstrated by shortening the time to first flatus pass and time to toleration of oral solid food compared with conventional fluid therapy when all studies were considered. Additionally, this meta-analysis investigated that GDFT combined with alpha-1 adrenergic receptor agonists reduced several postoperative complication rates, including myocardial ischemia, respiratory infection, respiratory support, PONV, and wound infection. However, it did not identify the beneficial effects of the intervention on short-term mortality and other complications associated with the urinary, coagulation, and neurological systems.

ERAS has been widely used in surgical treatment in recent years. It emphasizes the significance of avoiding tissue edema caused by volume overload throughout the perioperative period [53]. GDFT based on functional hemodynamic parameters also facilitates maintaining proper and effective intravascular volume. Although GDFT, which reduced postoperative complications and shortened LOS [28, 31, 34, 37, 38, 50], was widely used during the perioperative period, several studies demonstrated that GDFT was not associated with improved postoperative outcomes [9, 26, 27, 45].

Many anesthetics can cause vasodilation and reduce cardiac function. These effects are more obvious in elderly patients. Anesthesia and surgery may disturb microcirculation and induce systematic inflammation. They eventually lead to an imbalance between oxygen delivery and consumption in vital organs, increasing the risk of perioperative acute organ injury and long-term mortality [54–56]. Alpha-1 adrenergic receptor agonists are vasoactive drugs that can protect against vasodilation effects of anesthetics. When combined with GDFT, the use of alpha-1 adrenergic receptor agonists maintains vital organ perfusion without over-reliance on fluids [11–13]. Thus, this meta-analysis confirmed that surgical patients, especially elderly patients, might potentially benefit from GDFT combined with alpha-1 adrenergic receptor agonists.

Norepinephrine can be used to treat anesthesia-induced vasodilatation by increasing systemic vascular resistance owing to its alpha-1 adrenergic properties. Additionally, norepinephrine has slight, but dose-dependent, β-adrenergic effects that might be beneficial to counteract pure vasoconstriction. Several scholars were concerned that the resulting vasoconstriction could deteriorate microcirculatory blood flow in the intestinal tract and kidneys. However, Hiltebrand et al. [57] confirmed that the treatment with norepinephrine during the perioperative period had no adverse effects on microcirculatory blood flow or tissue oxygen tension in the intestinal tract. This finding was consistent with the result of the present study indicating a significant reduction in overall complication rates in subgroups using norepinephrine as an intervention.

Enhanced recovery of GI function was an important part of ERAS, which was related to shortened LOS and reduced postoperative gastrointestinal complications. Resinger et al. [42] observed a strong positive effect of Doppler-guided GDFT combined with noradrenaline or phenylephrine on gastrointestinal perfusion during surgery, indicating a euvolemic status in these patients because the gut is one of the organs primarily affected by the redistribution of blood to the vital organs in early hypovolemia [58]. The aforementioned findings were consistent with the results of the present study, including the reduction in the time to first flatus, oral solid food, and postoperative PONV in patients undergoing noncardiac surgery.

In the present study, GDFT combined with the use of alpha-1 adrenergic agonists was not accompanied by a reduced risk of postoperative AKI compared with the conventional fluid therapy. This was most likely due to the unexpected high achievement rate of hemodynamic goals in the control group with no further improvement in patients in whom the GDFT algorithm was applied. Schimid et al. [44] found that the short-term postoperative renal outcome was influenced by body mass index, preoperative creatinine clearance, perioperative hypovolemia, and the use of hydroxyethyl starch; the last two were controllable factors. Legrand et al. [12] showed that vasoconstriction induced by the recommended dosages of alpha-1 adrenergic receptor agonists did not significantly threaten renal perfusion and microcirculatory blood flow at proper volume status and cardiac function. However, anesthesiologists should still pay attention to elderly patients, especially patients with previous renal insufficiency, and limit the use of colloids during the perioperative period.

Most alpha-1 adrenergic receptor agonists were short-term agents that usually required continuous infusion to maintain blood concentration. Their continuous infusion should follow the principle that they should be initiated at a small dose and gradually titrated to the optimal dose. When a dose higher than recommended was needed to maintain the targeted blood pressure, anesthesiologists should actively find the leading reason for circulatory disorders. For patients with cardiovascular diseases, an improper use of alpha-1 adrenergic agonists may lead to serious consequences due to increased load on the left or right heart.

With a number of recently published trials on this topic, this report was the first systematic review and meta-analysis to evaluate the effect of GDFT combined with the application of alpha-1 adrenergic agonists on postoperative recovery after noncardiac surgery and was based on a comprehensive search strategy. Additionally, this systematic review included 11 high-quality studies [9, 24, 29–32, 34, 40, 43–45] and 12 newly published studies [28, 32, 34, 36, 37, 42, 45–50] which could provide powerful evidence of timeliness. However, this study had some limitations. The first limitation was its high heterogeneity, which might be related to different types of surgery, including abdominal surgery, thoracic surgery, orthopedic surgery, and others. Surgery type may have different effects on postoperative complications. Second, the quality of outcome data presented in the included RCTs was variable. Although the subgroup and sensitivity analyses could reduce the heterogeneity, not all planned analyses could be performed due to data insufficiency, such as the subgroup analyses based on the age of patients, type of surgery, and type of alpha-1 adrenergic agonists in GI function recovery. Third, funnel plots, Begg’s test, and Egger’s test were conducted in this review, and only the publication bias of wound infection indicated significant evidence. The absence of significant asymmetry for publication bias for other outcomes did not mean that a publication bias was absent [59]. Fourth, outcome measures were not consistent across all studies, and only relevant data from included trials could be considered for this meta-analysis because of the limitation of pooled analysis. Although GI function recovery was regarded as a meaningful outcome following noncardiac surgery, only 4 of the 31 included RCTs provided data on this outcome. Finally, 61.2% of included RCTs had small sample sizes (< 100), leading to the lack of statistical power.

## Conclusions

This systematic review and meta-analysis of available evidence suggested that the use of GDFT combined with alpha-1 adrenergic agonists could improve postoperative recovery following noncardiac surgery, as demonstrated by shortening of LOS, reduction in postoperative complications, and earlier recovery of GI function. Nevertheless, adequately powered, high-quality RCTs are needed to address the shortcomings of this study.

## Additional files

Additional file 1: Search terms and number of studies found from a preliminary PubMed search. (PDF 53 kb)

Additional file 2: Risk-of bias-summary: review authors’ judgments about each risk-of-bias item for each included study. (PDF 6354 kb)

Additional file 3: Sensitive analysis for short-term mortality. The influence of individual studies on the pooled RR. (PDF 2101 kb)

Additional file 4: Publication funnel plots for short-term mortality. RR, Risk ratio. (PDF 651 kb)

Additional file 5: Sensitive analysis for length of hospital stay. The influence of individual studies on the pooled RR. (PDF 2064 kb)

Additional file 6: Publication funnel plots for the length of hospital stay. WMD, Weighted mean difference. (PDF 698 kb)

Additional file 7: Forest plot comparing length of hospital stay for patients receiving GDFT combined with alpha-1 adrenergic agonists versus control, divided by risk of bias: Low, unclear and high risk of bias. (PDF 242 kb)

Additional file 8: Sensitive analysis for overall postoperative complication rates. The influence of individual studies on the pooled RR. (PDF 1592 kb)

Additional file 9: Publication funnel plots for short-term mortality. RR, Risk ratio. (PDF 677 kb)

Additional file 10: Forest plot comparing overall complication rates for patients receiving GDFT combined with alpha-1 adrenergic agonists versus control, divided by (a) age of patients: patients aged < 65 years and patients aged ≥65 years and (b) type of alpha-1 adrenergic agonists: norepinephrine, phenylephrine, norepinephrine combined with phenylephrine, or metaraminol. (PDF 8281 kb)

## Abbreviations

AKI: Acute kidney injury
CI: Cardiac index
CO: Cardiac output
CVP: Central venous pressure
DO_2_I: Oxygen delivery index
ELWI: Extravascular lung water index
ERAS: Enhanced recovery after surgery
FTc: Corrected flow time
GDFT: Goal-directed fluid therapy
GEDI: Global end-diastolic index
GI: Gastrointestinal
GRADE: Grading of Recommendations, Assessment, Development and Evaluations
LOS: Length of hospital stay
MAP: Mean arterial pressure
MEDLINE: Medical Literature Analysis and Retrieval System Online
O_2_ER: Oxygen extraction ratio
PONV: Postoperative nausea and vomiting
PPV: Pulse pressure variation
PRISMA: Preferred Reporting Items for Systematic Reviews and Meta-Analyses
PVI: Pleth variability index
RCT: Randomized controlled trial
RR: Risk ratio
SV: Stroke volume
SVI: Stroke volume index
SVV: Stroke volume variation
WMD: Weighted mean difference
