# Supplementary material for: Effects of perioperative goal-directed fluid therapy combined with the application of alpha-1 adrenergic agonists on postoperative outcomes: a systematic review and meta-analysis
Source: BMC Anesthesiol. 2018 Aug 17;18:113. doi: 10.1186/s12871-018-0564-y (PMC6098606; doi:10.1186/s12871-018-0564-y)
Supplement: Supplementary file 1 — Search terms and number of studies found from a preliminary PubMed search. (PDF 53 kb) [file 12871_2018_564_MOESM1_ESM.pdf]

Additional file 1 Search terms and number of articles found from a preliminary PubMed search

Step	Searching terms	Articles found (n)*
#1	((((((((((("General Surgery"[Mesh]) OR "Surgical Procedures, Operative"[Mesh]) OR "Perioperative Period"[Mesh]) OR "Monitoring, Intraoperative"[Mesh]) OR "Intraoperative Period"[Mesh]) OR "Intraoperative Care"[Mesh]) OR "surgery" [Subheading]) OR surg*) OR intraoperati*) OR perioperati*) OR operati*)) NOT "Cardiac Surgical Procedures"[Mesh]	4007563
#2	(((((("Fluid Therapy"[Mesh]) OR (((((("Body Fluids"[Mesh]) OR "Hemodynamics"[Mesh]) OR fluid*) OR hemodynamic*)) AND ((((((therap*) OR load*) OR administrat*) OR manag*) OR maintain*) OR optimizat*) OR optimisat*)))) AND ((("Goals"[Mesh]) OR goals) OR goal))) OR GDFT) OR GDHT	9032
#3	((((((((((((((("Randomized Controlled Trials as Topic"[Mesh]) OR "Randomized Controlled Trial" [Publication Type]) OR "Controlled Clinical Trial" [Publication Type]) OR "Controlled Clinical Trials as Topic"[Mesh]) OR "Placebos"[Mesh]) OR "Random Allocation"[Mesh]) OR "Clinical Trial" [Publication Type]) OR "Clinical Trials as Topic"[Mesh]) OR random*) OR trial) OR trials) OR group) OR groups) OR placebo) OR placebos)) NOT (("Animals"[Mesh])NOT"Humans"[Mesh])	4269271
#4	#1 AND #2 AND #3	815

*Preliminary search conducted on 1 March 2018 at 08:20:50.
MeSH, Medical Subject Heading; tiab, title abstract text.
