# Supplementary material for: Effects of perioperative goal-directed fluid therapy combined with the application of alpha-1 adrenergic agonists on postoperative outcomes: a systematic review and meta-analysis
Source: BMC Anesthesiol. 2018 Aug 17;18:113. doi: 10.1186/s12871-018-0564-y (PMC6098606; doi:10.1186/s12871-018-0564-y)
Supplement: Supplementary file 2 — Risk-of bias-summary: review authors’ judgments about each risk-of-bias item for each included study. (PDF 6354 kb) [file 12871_2018_564_MOESM2_ESM.pdf]

	Random sequence generation (selection bias)	Allocation concealment (selection bias)	Blinding of participants and personnel (performance bias)	Blinding of outcome assessment (detection bias)	Incomplete outcome data (attrition bias)	Selective reporting (reporting bias)	Other bias
Bartha et al., 2013	+	+	−	−	+	+	+
Benes et al., 2015	+	+	+	+	+	?	+
Bisgaard, et al., 2013	+	?	+	+	+	+	?
Broch et al., 2016	+	?	?	?	+	+	+
Elgendy et al., 2017	+	+	+	+	+	+	?
Forget et al., 2010	+	?	?	+	+	+	+
Funk et al., 2015	+	+	?	+	+	+	+
Gan et al., 2002	+	+	−	+	+	+	+
Gómez-Izquierdo et al., 2017	+	+	+	+	+	+	+
Hand et al., 2016	+	?	−	−	?	+	+
Kaufmann et al., 2017	+	+	+	+	+	+	+
Kumar et al., 2015	+	?	?	?	+	?	?
Luo et al., 2017	+	+	?	?	+	+	+
Malbouisson et al., 2017	−	?	−	?	+	+	?
Mayer et al., 2010	+	+	+	+	+	+	+
Moppett et al., 2015	+	+	−	+	+	+	+
Peng et al., 2014	+	+	−	+	+	?	?
Pestaña et al., 2014	+	+	+	+	+	+	+
Pösö et al., 2014	?	?	−	−	+	+	+
Reisinger et al., 2017	+	?	+	+	+	+	?
Salzwedel et al., 2013	+	+	−	−	+	+	+
Scheeren et al.,2013	+	+	−	+	+	?	?
Schmid et al., 2016	+	?	+	+	+	+	+
Stens et al., 2017	+	+	+	+	+	+	+
Veelo et al., 2017	?	?	?	?	+	+	?
wagar et al., 2017	?	?	?	?	+	+	+
Weinberg et al., 2017	+	+	+	+	+	?	+
Wu et al., 2017	+	+	+	+	+	?	?
Xu et al., 2017	+	+	+	+	+	+	?
Zhang et al., 2013	+	+	?	?	+	+	?
Zheng et al., 2013	+	+	?	+	?	+	+
