# Supplementary figures and images for: Effects of perioperative goal-directed fluid therapy combined with the application of alpha-1 adrenergic agonists on postoperative outcomes: a systematic review and meta-analysis
Source: BMC Anesthesiol. 2018 Aug 17;18:113. doi: 10.1186/s12871-018-0564-y (PMC6098606; doi:10.1186/s12871-018-0564-y)

# Meta-analysis estimates, given named study is omitted

| Lower CI Limit

○ Estimate

| Upper CI Limit

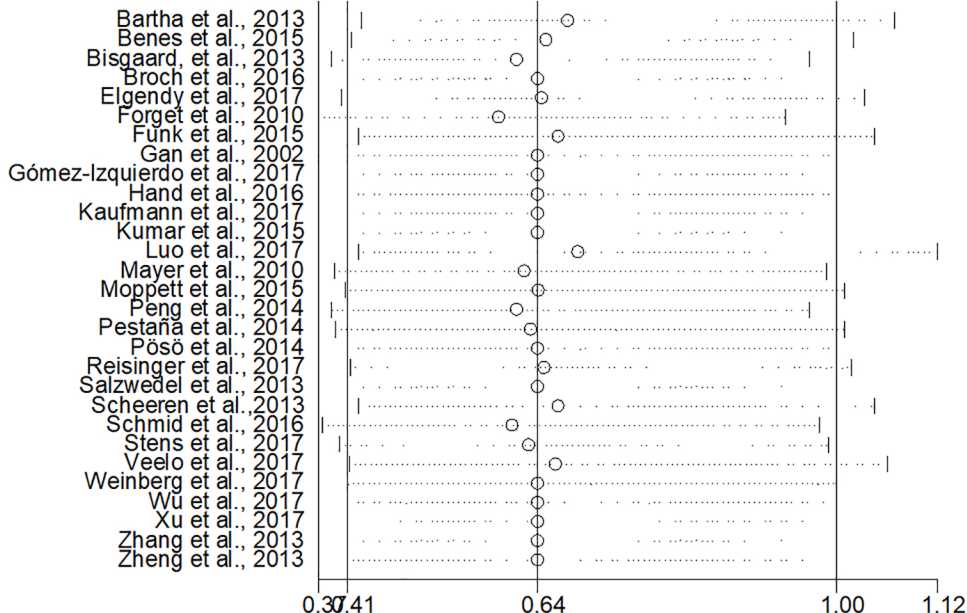

Supplement: Supplementary file 3 — Sensitive analysis for short-term mortality. The influence of individual studies on the pooled RR. (PDF 2101 kb) [file 12871_2018_564_MOESM3_ESM.pdf]

Funnel plot with pseudo 95% confidence limits

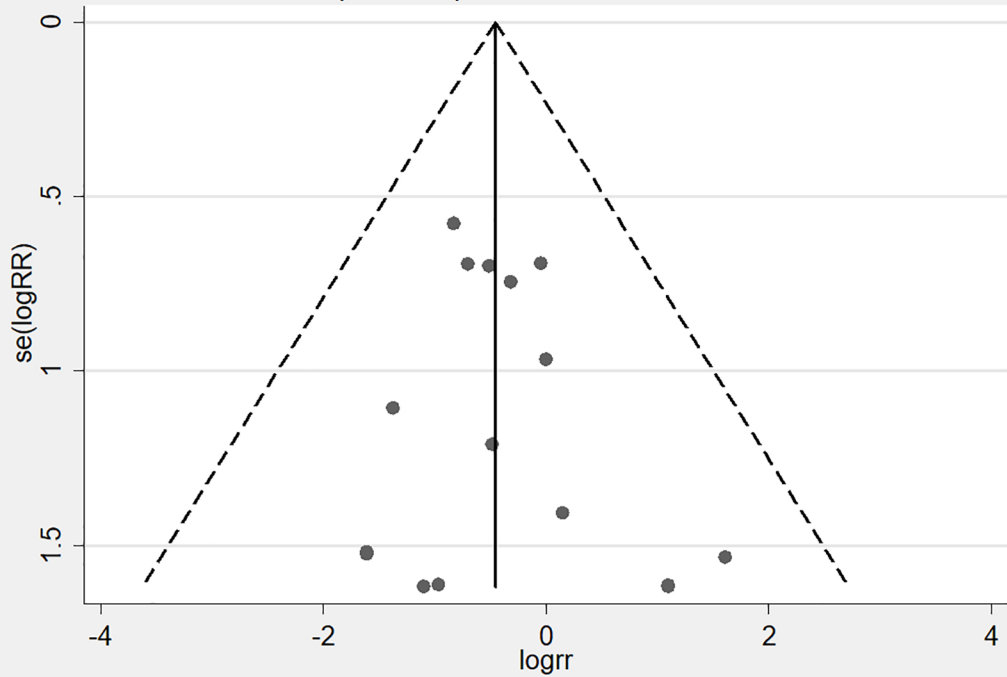

Supplement: Supplementary file 4 — Publication funnel plots for short-term mortality. RR, Risk ratio. (PDF 651 kb) [file 12871_2018_564_MOESM4_ESM.pdf]

# Meta-analysis estimates, given named study is omitted

| Lower CI Limit

○ Estimate

| Upper CI Limit

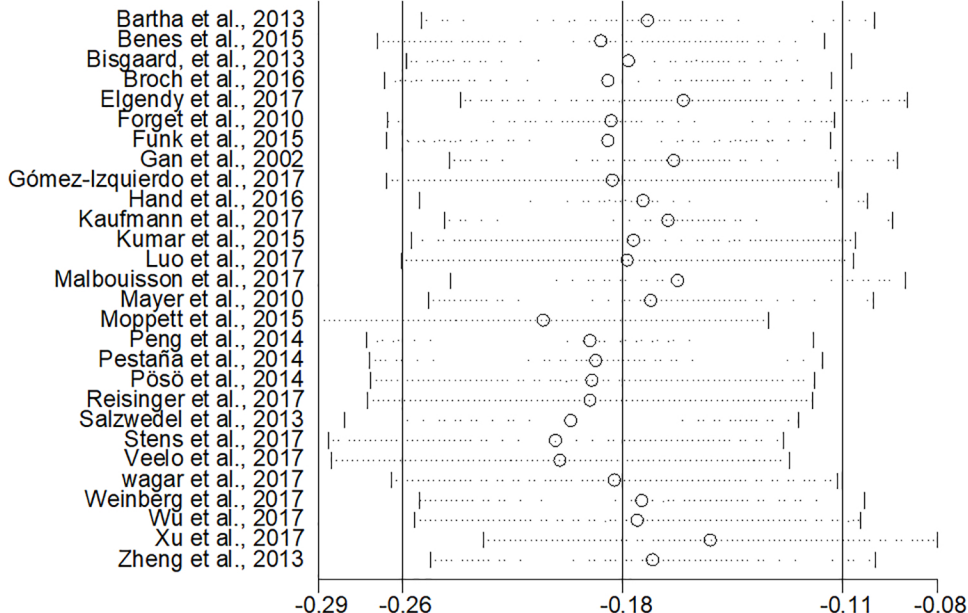

Supplement: Supplementary file 5 — Sensitive analysis for length of hospital stay. The influence of individual studies on the pooled RR. (PDF 2064 kb) [file 12871_2018_564_MOESM5_ESM.pdf]

Funnel plot with pseudo 95% confidence limits

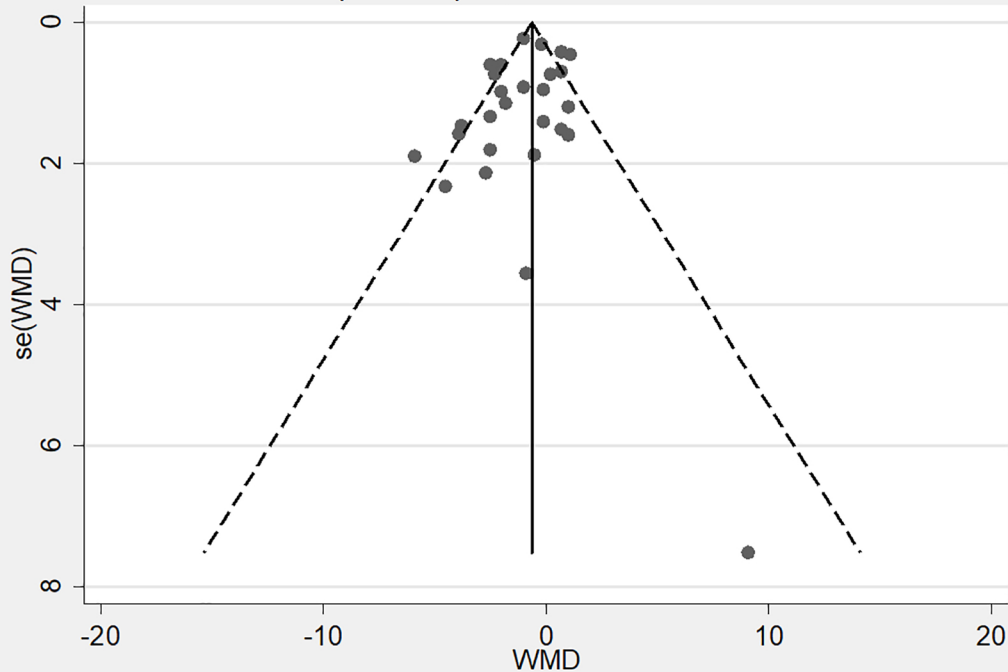

Supplement: Supplementary file 6 — Publication funnel plots for the length of hospital stay. WMD, Weighted mean difference. (PDF 698 kb) [file 12871_2018_564_MOESM6_ESM.pdf]

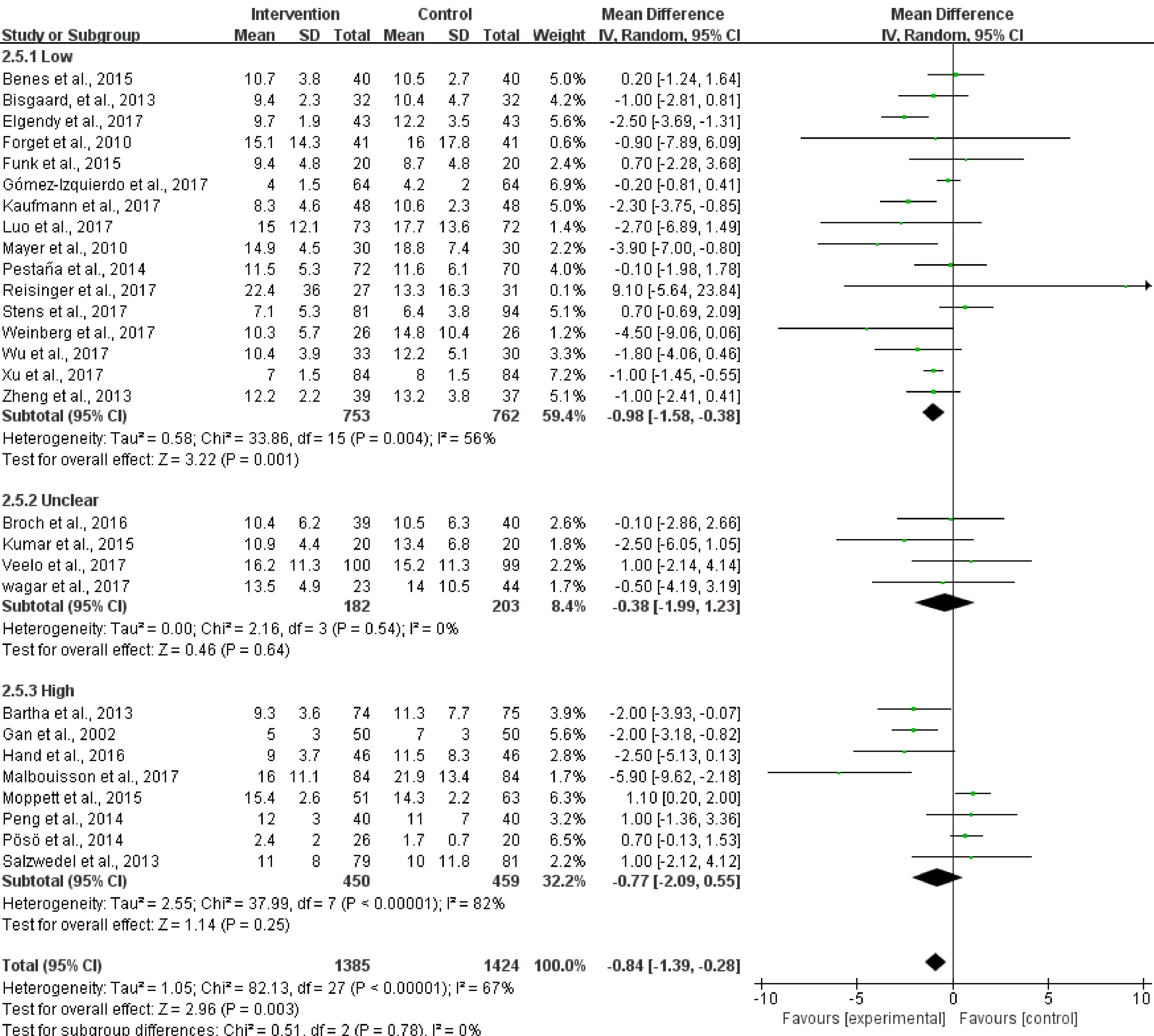

Supplement: Supplementary file 7 — Forest plot comparing length of hospital stay for patients receiving GDFT combined with alpha-1 adrenergic agonists versus control, divided by risk of bias: Low, unclear and high risk of bias. (PDF 242 kb) [file 12871_2018_564_MOESM7_ESM.pdf]

# Meta-analysis estimates, given named study is omitted

| Lower CI Limit

○ Estimate

| Upper CI Limit

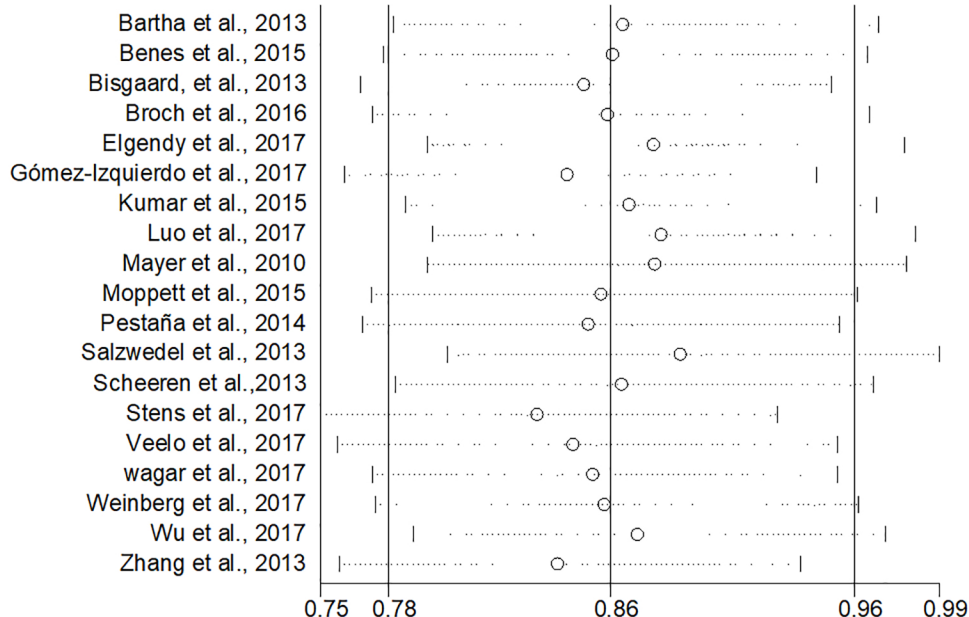

Supplement: Supplementary file 8 — Sensitive analysis for overall postoperative complication rates. The influence of individual studies on the pooled RR. (PDF 1592 kb) [file 12871_2018_564_MOESM8_ESM.pdf]

Funnel plot with pseudo 95% confidence limits

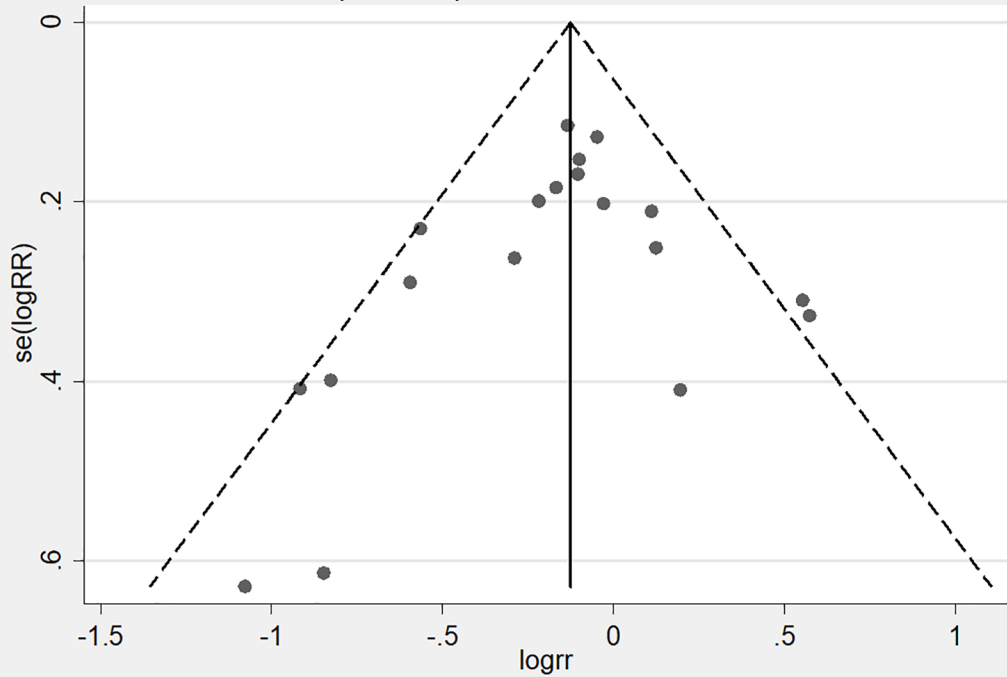

Supplement: Supplementary file 9 — Publication funnel plots for short-term mortality. RR, Risk ratio. (PDF 677 kb) [file 12871_2018_564_MOESM9_ESM.pdf]

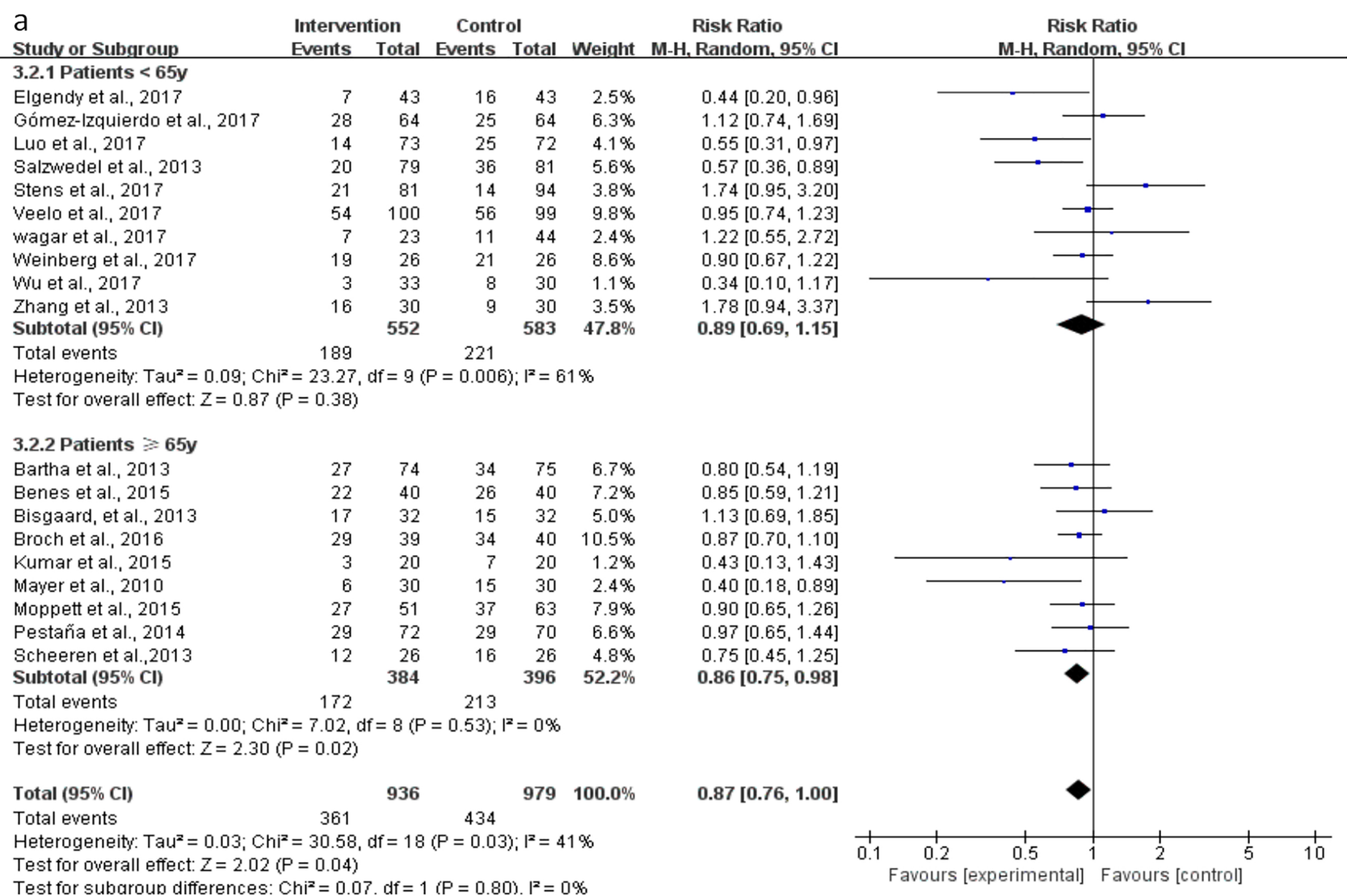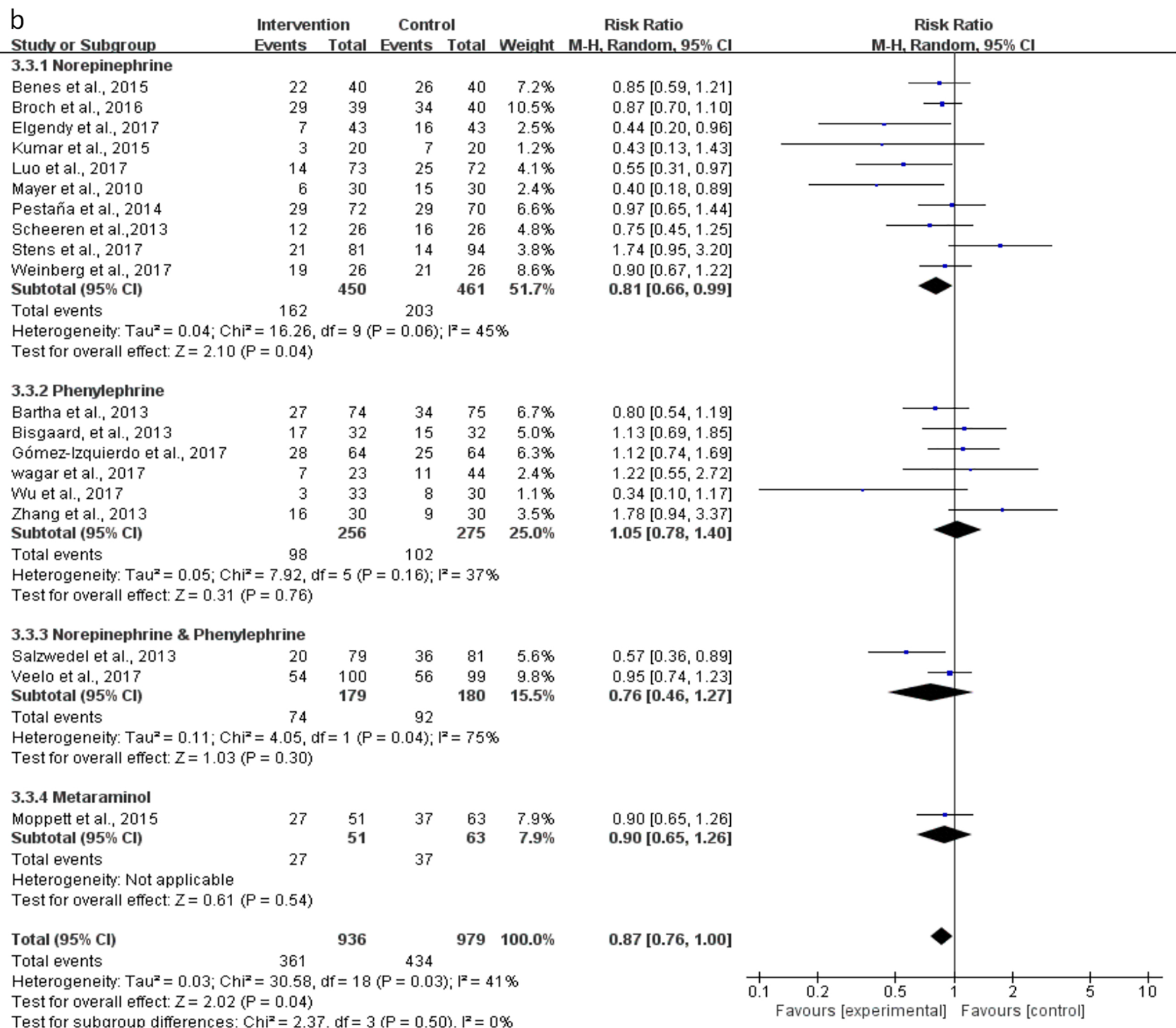

Supplement: Supplementary file 10 — Forest plot comparing overall complication rates for patients receiving GDFT combined with alpha-1 adrenergic agonists versus control, divided by (a) age of patients: patients aged < 65 years and patients aged ≥65 years and (b) type of alpha-1 adrenergic agonists: norepinephrine, phenylephrine, norepinephrine combined with phenylephrine, or metaraminol. (PDF 8281 kb) [file 12871_2018_564_MOESM10_ESM.pdf]
